# LC–MS/MS Method for Simultaneous Determination of Linarin and Its Metabolites in Rat Plasma and Liver Tissue Samples: Application to Pharmacokinetic and Liver Tissue Distribution Study After Oral Administration of Linarin

**DOI:** 10.3390/molecules24183342

**Published:** 2019-09-13

**Authors:** Yang Li, Chenxi Guang, Na Zhao, Xinchi Feng, Feng Qiu

**Affiliations:** 1 School of Chinese Materia Medica, Tianjin University of Traditional Chinese Medicine, Tianjin 301617, China; yangli940206@163.com (Y.L.); guangcx1994@163.com (C.G.); runningzn@163.com (N.Z.); 2 Tianjin State Key Laboratory of Modern Chinese Medicine, Tianjin University of Traditional Chinese Medicine, Tianjin 301617, China

**Keywords:** linarin, metabolites, LC–MS/MS, pharmacokinetics, liver tissue distribution

## Abstract

Linarin, a flavone glycoside, is considered to be a promising natural product due to its diverse pharmacological activities. Recently, it has been brought into focus for its potential to treat liver failure. In this study, a rapid and sensitive liquid chromatography electrospray-ionization tandem mass spectrometry (LC–MS/MS) method was developed and validated for the simultaneous determination of linarin and its three metabolites (acacetin, apigenin, and *p*-hydroxy benzaldehyde) in plasma and liver tissue samples of normal rats and rats with d-galactosamine (d-GalN)-induced liver injury. After liquid–liquid extraction (LLE) with ethyl acetate, chromatographic separation of the four analytes was achieved using an ACQUITY UPLC BEH-C18 (1.7 μm, 2.1 × 50 mm) with a mobile phase of 0.01% formic acid in methanol and 0.01% formic acid at a flow rate of 0.3 mL/min. The detection was accomplished on a tandem mass spectrometer via an electrospray ionization (ESI) source by multiple reaction monitoring (MRM) in the negative ionization mode. The method had a good linearity over the concentration range of 1.00–200 ng/mL for linarin and its metabolites. The validated method was successfully applied to the pharmacokinetic and liver tissue distribution study of linarin and its metabolites after a single oral administration of linarin (90 mg/kg) to rats.

## 1. Introduction

**Figure 1:**
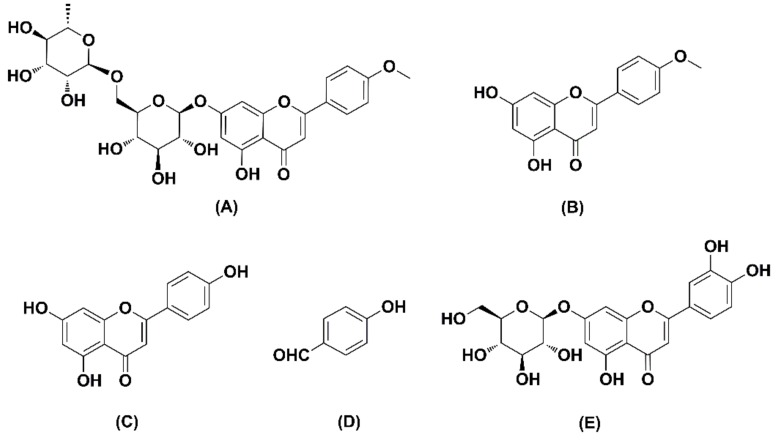
Structures of linarin (**A**), acacetin (**B**), apigenin (**C**), *p*-hydroxy benzaldehyde (**D**), and luteolin-7-*O*-glucopyranoside (**E**).

Linarin (acacetin-7-*O*-β-d-rutinoside) (Figure 1) is an active flavonoid glycoside isolated from *Chrysanthemum indicum* L., which has been discovered to possess diverse pharmacological activity [1,2,3,4,5,6,7]. As the main active component of *Chrysanthemum indicum* L, linarin not only retains the main antipyretic, analgesic, and anti-inflammatory effects of *Chrysanthemum indicum* L [1], but also exhibits a variety of pharmacological activities, such as sedative, neuroprotective, anti-apoptotic, and acetylcholinesterase and aldose reductase inhibitory activities [2,3,4,5,6,7]. Among them, the hepatoprotective effect of linarin has been investigated by increasingly more researchers [8,9,10]. Studies have shown that linarin can exert an hepatoprotective effect by inhibiting inflammatory reaction and cell apoptosis [3]. Moreover, a recent research carried out by Kim et al. [9] demonstrated the protective effect of linarin against severe hepatic failure in mice induced by d-galactosamine (d-GalN)/lipopolysaccharide (LPS), which suggested the potential of linarin in clinical applications for the treatment of liver injury.

However, one of our previous studies revealed that linarin shows low bioavailability (0.47%) after oral administration, which suggests that linarin could be extensively metabolized after oral administration, and the metabolites of linarin could still show various pharmacological activities [11]. Acacetin, apigenin, and *p*-hydroxy benzaldehyde (Figure 1) have been identified as the metabolites of linarin [12]. We found that these three metabolites showed a protective effect against severe d-GalN-induced hepatic failure in rats (data not shown). Up to now, the pharmacokinetic study of linarin had been extensively reported [11,13,14,15,16]; however, no pharmacokinetic study of its metabolites is available. Thus, further studies to elucidate the pharmacokinetics and liver tissue distribution of linarin and its active metabolites are still needed and will be helpful for a better understanding of the pharmacological effects of linarin.

Several analytical methods have been proposed for the determination of linarin in biological samples using liquid chromatography tandem mass spectrometry (LC–MS/MS) [11,14,15,16]. However, to the best of our knowledge, no analytical method has been reported for the simultaneous determination of linarin and its three active metabolites (acacetin, apigenin, and *p*-hydroxy benzaldehyde). In the study conducted by Zhang et al., an LC-MS/MS method for the simultaneous determination of seven flavonoids, including linarin, acacetin, and apigenin, was reported [15]. However, a large of volume rat plasma (100 μL) was required for sample preparation to achieve lower limits of quantification (LLOQs) of 1.0 ng/mL for linarin, acacetin, and apigenin. As a result, the cumulative blood collection volume exceeded 10% of an animal’s total blood volume. Therefore, in this study, a sensitive and selective LC–MS/MS method for the simultaneous determination of linarin and its three active metabolites in rat plasma and liver tissue was developed and validated. Only 20 μL of plasma or liver tissue homogenate was required in this method, and the method was successfully applied to pharmacokinetic and liver tissue distribution studies of linarin and its metabolites in both normal and d-GalN-induced liver injury rats after the oral administration of linarin.

## 2. Results and Discussion

### 2.1. Method Development

To obtain selectivity and sensitivity for all analytes, several mass and chromatographic spectrometric conditions were optimized. During the preliminary research, positive and negative ionization modes were compared to obtain high sensitivity, and the negative ionization mode was finally considered. Different fragmentors and collision energies were tested, and the optimized conditions for analytes and internal standard (IS) were selected as shown in Table 1. In addition, the optimum gas temperature, gas flow rate, nebulizer, and capillary voltage in the electrospray ionization (ESI) source were also established. Optimization of the mobile phase was conducted by comparing methanol-water and acetonitrile-water, and no significant difference was observed. Considering the high price and high toxicity of acetonitrile, methanol-water was finally selected for our study. Additionally, the mobile phase with the addition of different amounts of formic acid (0.01%, 0.05%, and 0.1%) was also evaluated. Finally, 0.01% formic acid and 0.01% formic acid in methanol were selected as the mobile phase in this study. An elution time of 13 min guaranteed that each analyte and internal standard (IS) had been completely eluted and separated from each other.

The purpose of sample extraction optimization is mainly to achieve high extraction recovery and low matrix effects in order to improve the sensitivity and reliability of LC–MS/MS analysis. Compared with protein precipitation, liquid–liquid extraction (LLE) was found to offer satisfactory recovery and low matrix effects in determining the concentrations of the analytes. Extractabilities of linarin and its metabolites from plasma with various organic solvents such as ethyl acetate and ethyl acetate–n-butyl alcohol (1:1, *v*/*v*) under neutral or acidic conditions were evaluated in our study. No significant difference in extraction efficiency was observed between these extraction solvents. However, more matrix interferences were observed when the plasma sample was acidified by the addition of formic acid before the extraction with organic solvents. Taking linarin as an example, a 1.7-fold increase in matrix interferences was observed when the plasma was extracted with ethyl acetate under acidic conditions compared with under neutral conditions. Finally, LLE with ethyl acetate was adopted because it yielded consistent recoveries for the analytes and IS, and fewer matrix interferences caused by the endogenous components in rat plasma.

The ideal IS for LC–MS/MS analysis is the isotope-labeled form of the analyte, which elutes at the same time as the analyte and can compensate for matrix effects or instrument instability [17]. Owing to the absence of commercially available isotope-labeled analytes, some structural analogs of the analytes, including icaritin, caffeic acid, and luteolin-7-*O*-glucopyranoside, were tested as IS in the present study. Under the above sample preparation conditions, icaritin showed low solubility, and the extraction recovery of caffeic acid was low. The retention time of luteolin-7-*O*-glucopyranoside was between that of the analytes, and the extraction recovery of luteolin-7-*O*-glucopyranoside was similar to that of linarin. Therefore, luteolin-7-*O*-glucopyranoside was selected as the IS in the present study. Additionally, linarin has been reported to be isolated from several plants, including *Flos chrysanthemi indici*, *Buddleja officinalis*, *Cirsium setosum*, *Mentha arvensis,* and *Buddleja davidii*. To the best of our knowledge, luteolin-7-*O*-glucopyranoside has not been reported to be present in the above-mentioned plants. Thus, the method developed in our study with 7-*O*-glucopyranoside as IS could also be applied to measure plasma levels of linarin and its metabolites after administration of plant extracts containing linarin.

### 2.2. Method Validation

#### 2.2.1. Selectivity

**Figure 2:**
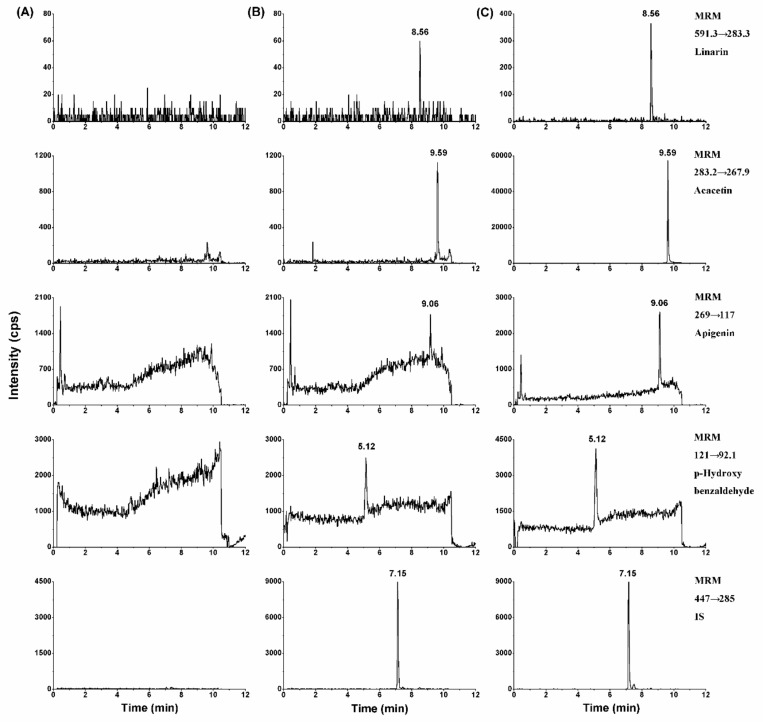
Multiple reaction monitoring (MRM) chromatograms of linarin, acacetin, apigenin, and *p*-hydroxy benzaldehyde from rat plasma. (**A**) Blank rat plasma; (**B**) blank plasma samples spiked with standard substance (1.00 ng/mL) and IS (50.0 ng/mL); (**C**) real plasma samples obtained from a rat following administration of 90 mg/kg linarin.

By comparing chromatograms of rat blank plasma samples with corresponding spiked plasma samples at lower limit of quantification (LLOQ) and rat plasma samples collected from rats after intragastric administration of linarin at 90 mg/kg, no endogenous substances interfered at the retention times of analytes and IS in all the blank samples. The retention times of linarin, acacetin, apigenin, *p*-hydroxy benzaldehyde, and IS were 8.56, 9.59, 9.06, 5.12, and 7.15 min, respectively (Figure 2).

#### 2.2.2. Analytical Curve and Lower Limit of Quantification

The analytical curves were linear over the concentration range of 1.00–200 ng/mL for the four analytes when performing weighted least square linear regression analysis with a weigh factor of 1/x^2^ in rat plasma. All correlation coefficients (r) were > 0.99, and the back-calculated concentrations of the calibration standards were within 15% of their nominal values. The LLOQs for linarin, acacetin, apigenin, and *p*-hydroxy benzaldehyde were established at 1.00 ng/mL. The detailed results are listed in Table 2.

#### 2.2.3. Accuracy and Precision

The inter- and intra-day accuracy and precision data of the determination of linarin and its metabolites in rat plasma are shown in Table 3. At each quality control (QC) level, the intra- and inter-day precisions (relative standard deviation, RSD) of these analytes were within 2.6–14.6%, and the accuracy (relative error, RE) ranged from −4.5% to 3.7%. All the values were within the acceptable range, indicating that the method is reliable and reproducible for the determination of analytes in rat plasma.

#### 2.2.4. Extraction Recovery and Matrix Effects

The extraction recovery and matrix effects of the analytes at three QC concentration levels and IS at 50.0 ng/mL were examined in this study (Table 3). The extraction recoveries of the four analytes in plasma at three QC concentration levels were within the range of 60.5–76.1%, and the recovery of IS was 48.9%. The matrix effects for the four analytes were found to be within the acceptable range of 88.1–107.9%, and the matrix effect of IS was 108.4%. These results reveal that no significant matrix effect was observed, and the LLE efficiency was acceptable in the current conditions.

#### 2.2.5. Stability

The freeze-thaw stability, long-term stability, short-term stability, and post-preparative stability results in plasma samples at two QC concentration levels are shown in Table 4. The RSD values ranged from 1.5% to 11.0%, and the RE values ranged from −10.0% to 11.5%. These results indicated that linarin and its metabolites were stable in rat plasma under the test conditions.

### 2.3. Application

The validated method was applied to investigate the pharmacokinetics and liver tissue distribution of linarin and its metabolites in rats after intragastric administration linarin (90 mg/kg).

#### 2.3.1. Pharmacokinetic Study

**Figure 3:**
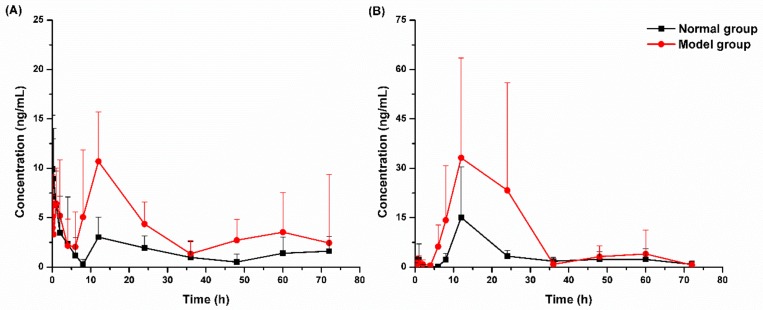
Plasma concentration–time curves of linarin (**A**) and acacetin (**B**) after oral administration of linarin in normal and model rats (*n* = 8).

After the oral administration of linarin, the metabolites apigenin and *p*-hydroxy benzaldehyde could only be detected in plasma at several time points (Tables S1 and S2). Thus, the plasma concentration–time curves for apigenin and *p*-hydroxy benzaldehyde were incomplete and no pharmacokinetic parameters for apigenin and *p*-hydroxy benzaldehyde were calculated. The main pharmacokinetic parameters of linarin and acacetin calculated by the non-compartmental model (DAS 3.2.8) are listed in Table 5. The mean plasma concentration–time curves of linarin and acacetin are shown in Figure 3.

Pharmacokinetic data showed that linarin and its three metabolites could all be absorbed into the blood, and acacetin was the main metabolite of linarin in the blood circulation after oral administration. After normal rats were given linarin intragastrically, linarin was rapidly absorbed, and the peak concentration was reached about 7 min later and was about 14 ng/mL. The concentration of acacetin, the main metabolite, reached its peak at about 12 h. In model rats, the absorption kinetics of linarin and the main metabolite acacetin were significantly different from that of the normal group. In model rats, the peak time of linarin was delayed, the peak concentration of acacetin was about 41 ng/mL, and the in vivo exposure of linarin and acacetin (AUC_0–t_) significantly increased. The significant differences in the pharmacokinetic profiles of linarin and its metabolites observed between normal and liver injury rats suggested that the pathological state of liver injury may affect the absorption of linarin and its metabolites.

#### 2.3.2. Liver Tissue Distribution

**Figure 4:**
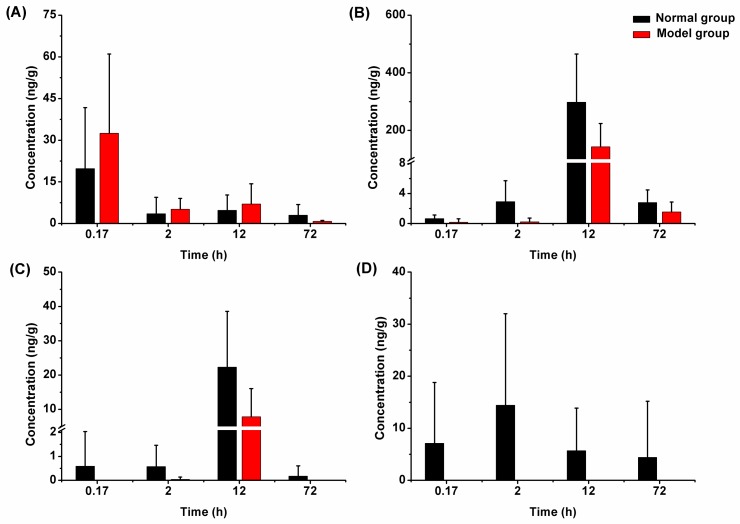
Liver tissue distribution of linarin (**A**), acacetin (**B**), apigenin (**C**), and *p*-hydroxy benzaldehyde (**D**) in normal and model rats after oral administration of linarin (*n* = 6).

The concentrations of linarin and its metabolites in liver tissues of normal and model rats determined at different time points are shown in Figure 4. The results showed that linarin and its metabolites were distributed in liver tissues of both model rats and normal rats. The highest levels of apigenin and acacetin in liver tissues were observed at 12 h after administration, while the highest level of *p*-hydroxy benzaldehyde was observed at 2 h after administration. Moreover, the distribution profiles exhibited an obvious downward trend, and no long-term accumulation was observed. In the comparison between normal rats and model rats, linarin and its metabolites showed significant differences, indicating that liver injury may affect the distribution process of linarin in the liver.

## 3. Materials and Methods

### 3.1. Chemicals and Reagents

Linarin, acacetin, and apigenin (purity >98%) were obtained from Shanghai Winherb Medical Technology Co. Ltd. (Shanghai, China). *p*-Hydroxy benzaldehyde (purity >98%) and LC/MS-grade formic acid were purchased from Shanghai Macklin Biochemical Co. Ltd. (Shanghai, China). Luteolin-7-*O*-glucopyranoside (IS, Figure 1) was isolated from the whole plant of *Phyhalis alkekengi* var. *franchetii* (collected from Tianjin, China) in the Laboratory of Tianjin University of Traditional Chinese Medicine (Tianjin, China). LC/MS-grade methanol was purchased from Fisher Scientific (Pittsburgh, PA, USA). Ultra-pure water was purchased from A.S. Watson Group Ltd. (Hong Kong, China). Other chemicals were of analytical grade.

### 3.2. Animals

All animal experiments were approved by the Animal Ethics Committee of Tianjin University of Traditional Chinese Medicine and carried out according to the Guide for the Care and Use of Laboratory Animals (National Institutes of Health). Male Sprague-Dawley rats weighting 180–220 g were obtained from the Beijing Military Medical Science Academy of the PLA (Beijing, China). In total, 64 rats were used in this study, and the rats were housed under controlled conditions (temperature 22 ± 2 °C, relative humidity 60 ± 5%) at a 12/12-h day/night cycle. Standard laboratory food and water were provided for the rats. The rats were fasted for at least 12 h before the experiments with free access to water.

### 3.3. Chromatographic and Mass Spectrometry Conditions

The LC–MS/MS system consisted of a Waters ultra-high-performance liquid chromatography ACQUITYTM I-Class system coupled to an AB SCIEX 5500 QTRAP triple quadrupole mass spectrometer. Chromatographic separation was achieved on an ACQUITY UPLC BEH-C18 (1.7 μm, 2.1 × 50 mm) with a mobile phase of (A) 0.01% formic acid and (B) 0.01% formic acid in methanol at a flow rate of 0.3 mL/min. The gradient elution program was as follows: 0–4.0 min, 10% B; 4.0–6.0 min, 10%–40% B; 6.0–8.0 min, 40%–55% B; 8.0–9.5 min, 55%–95% B; 9.5–10.5 min, 95% B; 10.5–10.6 min, 95%–10% B; 10.6–13 min, 10% B. The column was set at indoor temperature, whereas the auto-sampler tray was maintained at 12 ± 2 °C, and the sample injection volume was 2 μL for analysis.

The mass spectrometer was operated in the negative ESI mode, and the multiple reaction monitoring (MRM) mode was used for quantitation. The optimized MS parameters were designed as follows: curtain gas, 30.00 psi; collision gas, 10.00 psi; ion spray voltage, −4500.00 V; temperature, 500.0 °C; ion source gas 1, 50.00 psi; ion source gas 2, 50.00 psi. MS parameters of the analytes and IS are shown in Table 1. Data acquisition and quantitation were carried out using Analyst version 1.6.2 (Applied Biosystems/MDS SCIEX).

### 3.4. Preparation of Calibration Standard and Quality Control Samples

The stock solutions (stored at −20 °C) of linarin, acacetin, apigenin, *p*-hydroxy benzaldehyde, and IS at a concentration of 10.0 mg/mL were dissolved in DMSO. Then, an appropriate amount of the four stock solutions was mixed and diluted with 50% methanol to obtain a series of mixed working solutions at concentrations of 1.00–200 ng/mL for the analytes. A quantity of IS stock solution was diluted with 50% methanol-water to produce the IS solution with a concentration of 50.0 ng/mL. QC working solutions at concentrations of 3.00, 25.0, and 160 ng/mL were obtained from a separately prepared 10.0 mg/mL stock solution of analytes and IS.

Calibration standards were prepared by adding 20 μL of the working solutions to 20 μL blank rat plasma or tissue homogenate to create a set of analytical curves (1.00, 2.00, 5.00, 10.0, 25.0, 50.0, 100, 200 ng/mL). QC samples were prepared by the same procedure as above at concentrations of 3.00, 25.0, and 160 ng/mL.

### 3.5. Sample Preparation

LLE with ethyl acetate was utilized for sample preparation. All samples stored at −80 °C were thawed at room temperature. An aliquot of 20 μL rat plasma or liver tissue homogenate, 20 μL of IS solution, and 20 μL 50% methanol were added into a centrifuge tube, and the mixture was then vortexed for 1 min. The mixture was extracted with 200 μL of ethyl acetate by vortex-mixing for 5 min and shaking on an orbital shaker for 30 min. After centrifugation at 14,000 rpm for 5 min, 200 μL supernatant layer was transferred to a clean tube and evaporated to dryness under a gentle stream of nitrogen at indoor temperature. The residue was reconstituted with 200 μL 50% methanol and centrifuged at 14,000 rpm for another 5 min.

### 3.6. Method Validation

The method was validated for specificity, linearity, accuracy, precision, extraction recovery, matrix effects, and stability in reference to the U.S. Food and Drug Administration (FDA) Bioanalytical Method Validation (Food and Drug Administration, 2018) [18].

The specificity was evaluated by comparing chromatograms of blank plasma samples from six different rats with samples of corresponding spiked plasma at the LLOQ and plasma after oral administration of linarin at 90 mg/kg.

The linearity of the method was evaluated by analyzing calibration standards in duplicate at each concentration level over 3 consecutive days. The analytical curves were constructed by plotting the peak area ratio of analytes to IS (y) versus the nominal analyte concentrations (x), and fitted by a weighted (1/x^2^) least square regression. The curves should have a correlation coefficient (*r*) of at least >0.99. The LLOQ was defined as the lowest concentration on the analytical curve with a RSD less than 20% and a deviation from the nominal concentration within ± 20% by six replicate analyses.

To determine intra- and inter-day precision and accuracy, six sets of spiked QC samples at low, medium, and high concentration levels were prepared and analyzed on three consecutive runs. The intra- and inter-day precisions were expressed as RSD, which should not exceed 15%. The accuracy was expressed in terms of the RE, which was required to be within ± 15%.

The extraction recoveries were measured by comparing peak areas of regular QC samples in six replicates with those in post-extracted blank plasma samples spiked at corresponding concentrations. The matrix effect was calculated by comparing the peak areas of the analytes obtained from the post-extraction spiked samples from six different replicates to those from the QC working solutions (i.e., 20 μL of QC working solutions and 20 μL of IS working solution added with 160 μL of 50% methanol) at the respective QC level. The post-extraction spiked samples were prepared as follows: 40 μL of 50% methanol was added to an aliquot of 20 μL blank plasma. The mixtures were extracted with 200 μL of ethyl acetate by vortex-mixing for 5 min and shaking on an orbital shaker for 30 min. After centrifugation at 14,000 rpm for 5 min, 200 μL supernatant layer was transferred to another clean tube. An aliquot of 20 μL QC working solutions and 20 μL IS working solution were added to the supernatant followed by evaporation to dryness. Then, the residue was reconstituted with 200 μL 50% methanol and centrifuged at 14,000 rpm for another 5 min. The matrix effect should be within 85–115%. The extraction recovery and matrix effects of IS were also determined at 50.0 ng/mL in the same way.

The stability of the analytes was evaluated by analyzing triplicates of QC samples at low and high concentration levels. The short-term stability was assessed by analyzing QC samples stored at room temperature for 4 h and post-preparative QC samples stored in the auto-sampler (12 °C) for 24 h. The long-term stability was assessed by evaluating the QC samples at −20 °C for 30 days. For freeze-thaw stability, QC samples were subjected to three complete freeze-thaw cycles.

### 3.7. Pharmacokinetic and Tissue Distribution

The method was applied to determine the pharmacokinetic and liver tissue distribution of linarin and its metabolites in rat plasma or liver tissue homogenate.

#### 3.7.1. Pharmacokinetic Study

Since linarin could not be dispersed in 0.5% CMC-Na uniformly, a homogeneous liquid preparation of linarin was prepared for oral administration in the pharmacokinetic study. Linarin showed good solubility in *N*,*N*-dimethylformamide, which was thus used as solvent. However, *N*,*N*-dimethylformamide showed hepatotoxicity after oral administration; therefore, several other excipients were added into the liquid preparation to dilute the concentration of *N*,*N*-dimethylformamide. Finally, the mixture of *N*,*N*-dimethylformamide–1,2-propanediol–0.5% CMC-Na–polyethylene glycol 400 (3:3:4:10, *v*/*v*/*v*/*v*) was selected.

Sixteen male Sprague–Dawley rats were randomly divided into two groups, and eight rats in each group were given 600 mg/kg d-GalN or the corresponding volume of saline by intraperitoneal injection. After 24 h, the rats were intragastrically given linarin prepared in *N*,*N*-dimethylformamide–1,2-propanediol–0.5% CMC-Na–polyethylene glycol 400 (3:3:4:10, *v*/*v*/*v*/*v*) at a dose of 90 mg/kg. Approximately 150 μL of blood samples were collected into EDTA-Na_2_ centrifuge tubes at 0, 0.03, 0.08, 0.17, 0.33, 1, 2, 4, 6, 8, 12, 24, 36, 48, 60, and 72 h. After centrifugation at 5000 rpm for 10 min, the supernatant was transferred into the clean centrifuge tube and stored at −20 °C before analysis.

#### 3.7.2. Liver Tissue Distribution Study

Forty-eight Sprague–Dawley rats were randomly divided into two groups: normal group and model group. The process of modeling and administration was the same as that used for the pharmacokinetic study. Six rats of each group were sacrificed at 0.17, 2, 12, and 72 h after intragastric administration of linarin at a dose of 90 mg/kg, respectively. Liver samples were collected and rinsed rapidly with physiological saline solution to remove the blood or content, and were then blotted on filter paper. Small slices of tissues (1 g) were individually homogenized in 50% methanol-water (3 mL) (1:3, *w*/*v*), and then centrifuged at 14,000 rpm for 5 min. The supernatant was separated and stored at −20 °C until analysis.

## 4. Conclusions

In this study, a sensitive LC–MS/MS method was developed and validated for the first time for the simultaneous determination of linarin and its three metabolites (acacetin, apigenin, and *p*-hydroxy benzaldehyde) in rat plasma and liver tissue samples. This method required a simple LLE procedure, and the total run time was 13 min per sample. The method was successfully applied to the pharmacokinetic and liver tissue distribution study of linarin and its metabolites in rats. This is also the first simultaneous study of linarin, acacetin, apigenin, and *p*-hydroxy benzaldehyde in terms of pharmacokinetics and liver tissue distribution after a single oral administration of linarin. Our findings will be helpful in the application of linarin in clinical therapy.

## Figures and Tables

**Table 1:** Mass scan method parameters of analytes and internal standard (IS).

Compounds	Q1	Q3	DP (V)	EP (V)	CE (V)	CXP (V)
Linarin	591.3	283.3	−120	−10	−22	−17
Acacetin	283.2	267.9	−100	−10	−30	−17
Apigenin	269	117	−45	−10	−45	−17
*p*-Hydroxy benzaldehyde	121	92.1	−108	−10	−35	−17
Luteolin-7-*O*-glucopyranoside (IS)	447	285	−40	−10	−37	−17

Abbreviations: DP, declustering potential; EP, entrance potential; CE, collision energy; CXP, collision cell exit potential.

**Table 2:** Analytical curves, correlation coefficient (*r*), linear range, and lower limit of quantification (LLOQ) of linarin, acacetin, apigenin, and *p*-hydroxy benzaldehyde.

Compounds	**Analytical Curves**	*r*	**Linear Range** (ng/mL)	LLOQ (ng/mL)	RSD (%)	RE (%)
Linarin	y = 0.00116x + 0.0011	0.9966	1.00–200	1.00	9.1	−4.1
Acacetin	y = 0.171x + 0.0921	0.9951	1.00–200	1.00	3.6	5.2
Apigenin	y = 0.0875x + 0.0366	0.9974	1.00–200	1.00	6.4	−0.6
*p*-Hydroxy benzaldehyde	y = 0.0767x + 0.1950	0.9955	1.00–200	1.00	7.2	3.1

**Table 3:** Intra-day/inter-day accuracy, precision, extraction recovery, and matrix effects of linarin, acacetin, apigenin, and *p*-hydroxy benzaldehyde in rat plasma.

Compounds	Added Concentration	Found Concentration	Intra-Day	Inter-Day	Accuracy	Recovery	Matrix Effect
	(ng/mL)	(ng/mL)	RSD (%)	RSD (%)	RE (%)	(%)	(%)
Linarin	3.00	2.97 ± 0.29	9.3	13.3	−0.9	60.9 ± 8.3	88.1 ± 7.8
	25.0	24.3 ± 2.35	8.0	14.6	−2.9	62.0 ± 6.8	102.8 ± 3.3
	160	155 ± 12.1	7.9	7.3	−3.2	60.5 ± 4.7	94.1 ± 2.0
Acacetin	3.00	2.87 ± 0.18	6.6	4.2	−4.5	76.1 ± 3.6	106.8 ± 5.5
	25.0	24.8 ± 0.94	3.3	6.4	−0.8	72.7 ± 1.3	104.1 ± 3.1
	160	163 ± 5.30	3.3	2.9	2.0	73.9 ± 1.6	104.1 ± 1.0
Apigenin	3.00	2.89 ± 0.22	8.0	4.3	−3.8	71.3 ± 3.8	105.4 ± 6.5
	25.0	24.4 ± 1.06	2.6	10.6	−2.5	72.4 ± 1.3	104.1 ± 1.9
	160	161 ± 7.86	3.1	11.4	0.4	74.0 ± 1.8	102.6 ± 1.7
*p*-Hydroxy benzaldehyde	3.00	2.97 ± 0.23	7.2	6.8	−0.9	61.0 ± 2.4	107.9 ± 7.8
	25.0	24.9 ± 1.39	4.8	9.8	−0.4	68.3 ± 2.7	101.9 ± 3.0
	160	166 ± 10.9	4.0	14.5	3.7	63.6 ± 1.0	104.3 ± 7.2
Luteolin-7-*O*-glucopyranoside (IS)	50.0	-	-	-	-	48.9 ± 1.4	108.4 ± 3.1

**Table 4:** Stability of linarin, acacetin, apigenin, and *p*-hydroxy benzaldehyde in rat plasma.

Compounds	Added Concentration	Post-Preparative Stability		Short-Term Stability		Freeze-Thaw Stability		Long-Term Stability	
	(ng/mL)	RE (%)	RSD (%)	RE (%)	RSD (%)	RE (%)	RSD (%)	RE (%)	RSD (%)
Linarin	3.00	7.0	3.7	5.3	8.9	−7.3	7.9	9.7	4.6
	160	−8.5	5.8	−8.5	1.5	−3.5	9.1	2.3	4.8
Acacetin	3.00	−7.0	3.9	−8.0	7.6	−1.3	5.7	−5.0	2.5
	160	3.3	5.0	7.9	6.4	4.4	2.4	2.9	2.3
Apigenin	3.00	6.0	11.0	−10.0	0.7	−7.0	3.9	−3.3	4.8
	160	−4.8	3.5	10.6	6.7	6.0	1.4	4.2	0.7
*p*-Hydroxy	3.00	7.7	4.0	11.3	3.3	−4.3	9.1	−7.0	3.2
benzaldehyde	160	0.4	6.1	7.3	4.2	11.5	3.7	9.4	3.0

**Table 5:** Pharmacokinetic parameters of linarin and acacetin after a single oral administration of linarin in normal rats and model rats.

Parameters	Linarin		Acacetin	
	Normal Rats	Model Rats	Normal Rats	Model Rats
t_1/2_z (h)	36.9 ± 21.8	37.4 ± 27.9	16.5 ± 3.7	11.0 ± 6.1
Tmax (h)	0.12 ± 0.10	8.65 ± 5.17	15.13 ± 13.83	12.25 ± 5.28
Cmax (μg/L)	14.40 ± 4.37	13.85 ± 4.47	16.78 ± 14.30	41.65 ± 30.77
AUC_0–t_ (μg/L·h)	174 ± 54	352 ± 99	282 ± 116	839 ± 682
AUC_0–∞_ (μg/L·h)	250 ± 52	367 ± 51	340 ± 128	919 ± 658
Vz/F (L/kg)	22517 ± 14103	9077 ± 4335	11233 ± 7029	2595 ± 2117
CLz/F (L/h/kg)	335 ± 145	225 ± 77	322 ± 187	149 ± 91

Abbreviations: t_1/2_z, half-life; Cmax, maximal observed plasma concentration; Tmax, time at which Cmax was observed; AUC_0-t_, area under the concentration-time curve from time 0 to t h postdose; AUC_0-∞_, area under plasma concentration-time profile from time 0 extrapolated to infinite time; Vz/F, apparent volume of distribution associated with the terminal phase; CLz/F, apparent clearance after oral administration.

## Supplementary Materials

The following are available online at https://www.mdpi.com/1420-3049/24/18/3342/s1, Table S1: Plasma concentrations of apigenin after oral administration of linarin in rats; Table S2: Plasma concentrations of *p*-hydroxy benzaldehyde after oral administration of linarin in rats.
