# Supplementary material for: LC–MS/MS Method for Simultaneous Determination of Linarin and Its Metabolites in Rat Plasma and Liver Tissue Samples: Application to Pharmacokinetic and Liver Tissue Distribution Study After Oral Administration of Linarin
Source: Molecules. 2019 Sep 13;24(18):3342. doi: 10.3390/molecules24183342 (PMC6766828; doi:10.3390/molecules24183342)
Supplement: Supplementary file 1 [file molecules-24-03342-s001.pdf]

Electronic Supplementary Information for

LC–MS/MS Method for Simultaneous Determination of Linarin and its Metabolites in Rat Plasma and Liver Tissue Samples: Application to Pharmacokinetic and Liver Tissue Distribution Study After Oral Administration of Linarin

Yang Li[a,b], Chenxi Guang[a,b], Na Zhao[a,b], Xinchi Feng[a,*] and Feng Qiu[a,b,*]

[a] School of Chinese Materia Medica, Tianjin University of Traditional Chinese Medicine, Tianjin 300193, China

[b] Tianjin State Key Laboratory of Modern Chinese Medicine, Tianjin University of Traditional Chinese Medicine, Tianjin 300193, China

Table of Contents

Supplemental Tables

Table S1. Plasma concentrations of apigenin after oral administration of linarin in rats S2

Table S2. Plasma concentrations of *p*-hydroxy benzaldehyde after oral administration of linarin in rats...S4

Table S1 Plasma concentrations of apigenin after oral administration of linarin in rats.

Groups	Time (h)	Concentration (ng/mL)								
		NO.1	NO.2	NO.3	NO.4	NO.5	NO.6	NO.7	NO.8	Mean ± SD
Normal rats	0.03	N.D.	N.D.	N.D.	1.37	10.20	N.D.	N.D.	1.30	1.61 ± 3.52
	0.08	1.11	4.92	1.34	N.D.	N.D.	2.81	N.D.	3.18	1.67 ± 1.81
	0.17	N.D.	1.07	N.D.	4.77	N.D.	N.D.	1.43	1.66	1.12 ± 1.64
	0.33	3.08	N.D.	2.15	1.02	1.90	2.06	5.64	1.54	2.17 ± 1.66
	1	1.09	N.D.	N.D.	2.12	1.24	2.34	1.17	1.93	1.24 ± 0.89
	2	N.D.	N.D.	N.D.	N.D.	N.D.	N.D.	N.D.	N.D.	0.00 ± 0.00
	4	N.D.	N.D.	N.D.	N.D.	N.D.	N.D.	N.D.	N.D.	0.00 ± 0.00
	6	N.D.	N.D.	N.D.	N.D.	N.D.	N.D.	N.D.	N.D.	0.00 ± 0.00
	8	N.D.	N.D.	N.D.	N.D.	N.D.	N.D.	N.D.	N.D.	0.00 ± 0.00
	12	3.44	N.D.	N.D.	N.D.	2.61	N.D.	N.D.	N.D.	0.76 ± 1.62
	24	N.D.	N.D.	N.D.	N.D.	N.D.	N.D.	N.D.	N.D.	0.00 ± 0.00
	36	N.D.	N.D.	1.04	N.D.	N.D.	N.D.	N.D.	N.D.	0.13 ± 0.37
	48	N.D.	N.D.	1.67	N.D.	N.D.	N.D.	N.D.	N.D.	0.21 ± 0.59
	60	N.D.	N.D.	N.D.	N.D.	N.D.	N.D.	N.D.	N.D.	0.00 ± 0.00
	72	N.D.	N.D.	N.D.	N.D.	N.D.	N.D.	N.D.	N.D.	0.00 ± 0.00

Model rats	0.03	N.D.	N.D.	1.41	N.D.	3.48	1.88	N.D.	1.81	1.07 ± 1.29
	0.08	N.D.	1.47	N.D.	N.D.	4.77	1.26	N.D.	N.D.	0.94 ± 1.67
	0.17	3.79	N.D.	2.24	N.D.	1.03	N.D.	N.D.	N.D.	0.88 ± 1.42
	0.33	N.D.	N.D.	3.97	N.D.	1.13	N.D.	N.D.	N.D.	0.64 ± 1.40
	1	N.D.	N.D.	N.D.	N.D.	N.D.	2.33	N.D.	N.D.	0.29 ± 0.82
	2	N.D.	N.D.	N.D.	3.13	N.D.	4.78	N.D.	N.D.	0.99 ± 1.88
	4	N.D.	N.D.	N.D.	N.D.	N.D.	N.D.	N.D.	N.D.	0.00 ± 0.00
	6	N.D.	N.D.	N.D.	4.15	N.D.	N.D.	N.D.	N.D.	0.52 ± 1.47
	8	N.D.	N.D.	1.38	5.85	N.D.	1.17	N.D.	N.D.	1.05 ± 2.02
	12	34.20	4.36	N.D.	2.91	2.37	1.05	1.20	N.D.	5.76 ± 11.59
	24	7.88	1.22	N.D.	N.D.	N.D.	1.32	N.D.	N.D.	1.30 ± 2.72
	36	2.76	N.D.	N.D.	N.D.	N.D.	N.D.	N.D.	N.D.	0.35 ± 0.98
	48	N.D.	N.D.	N.D.	N.D.	1.09	2.20	N.D.	N.D.	0.41 ± 0.82
	60	N.D.	1.29	N.D.	6.91	N.D.	N.D.	N.D.	N.D.	1.03 ± 2.42
	72	N.D.	N.D.	N.D.	1.05	N.D.	N.D.	7.17	N.D.	1.03 ± 2.51

Footnote: N.D. = not detected; SD = standard deviation.

Table S2 Plasma concentrations of *p*-hydroxy benzaldehyde after oral administration of linarin in rats.

Groups	Time (h)	Concentration (ng/mL)								
		NO.1	NO.2	NO.3	NO.4	NO.5	NO.6	NO.7	NO.8	Mean ± SD
Normal rats	0.03	N.D.	N.D.	N.D.	11.10	11.80	2.90	10.20	N.D.	4.50 ± 5.51
	0.08	19.90	N.D.	N.D.	N.D.	N.D.	14.10	N.D.	N.D.	4.25 ± 8.02
	0.17	N.D.	N.D.	N.D.	9.18	N.D.	N.D.	N.D.	N.D.	1.15 ± 3.25
	0.33	27.70	N.D.	N.D.	1.05	N.D.	N.D.	3.33	N.D.	4.01 ± 9.64
	1	N.D.	N.D.	N.D.	N.D.	N.D.	N.D.	15.10	N.D.	1.89 ± 5.34
	2	6.63	2.39	4.02	4.84	6.85	2.79	7.79	2.71	4.75 ± 2.11
	4	4.83	5.50	5.58	7.29	2.97	1.32	7.42	2.72	4.70 ± 2.20
	6	3.14	3.20	2.58	7.88	3.49	2.74	9.74	9.05	5.23 ± 3.09
	8	5.62	3.23	3.28	3.52	6.29	6.52	5.41	3.61	4.69 ± 1.40
	12	3.55	2.53	N.D.	2.68	3.14	3.12	N.D.	3.59	2.33 ± 1.48
	24	1.34	2.11	2.35	1.86	N.D.	3.20	1.89	1.76	1.81 ± 0.91
	36	1.74	N.D.	3.19	N.D.	1.04	N.D.	2.51	N.D.	1.06 ± 1.29
	48	1.05	N.D.	2.69	N.D.	N.D.	2.10	2.60	1.01	1.18 ± 1.16
	60	1.13	N.D.	3.59	N.D.	N.D.	2.99	N.D.	N.D.	0.96 ± 1.50
	72	1.56	7.30	13.50	N.D.	1.13	2.04	2.49	1.69	3.71 ± 4.51

Model rats	0.03	N.D.	10.80	11.40	N.D.	29.60	15.40	1.42	N.D.	8.58 ± 10.51
	0.08	5.88	N.D.	1.77	N.D.	25.70	22.90	N.D.	7.82	8.01 ± 10.49
	0.17	31.90	N.D.	N.D.	5.94	11.00	N.D.	8.26	8.42	8.19 ± 10.53
	0.33	2.81	18.30	14.20	N.D.	15.80	N.D.	6.47	7.04	8.08 ± 7.21
	1	3.25	N.D.	N.D.	2.90	N.D.	7.37	6.18	4.43	3.02 ± 2.89
	2	N.D.	7.24	N.D.	N.D.	N.D.	3.18	18.60	7.77	4.60 ± 6.54
	4	N.D.	N.D.	N.D.	N.D.	N.D.	7.23	6.67	3.19	2.14 ± 3.17
	6	N.D.	6.23	N.D.	N.D.	N.D.	3.06	N.D.	6.73	2.00 ± 2.96
	8	4.21	N.D.	N.D.	N.D.	N.D.	N.D.	3.07	3.99	1.41 ± 1.97
	12	85.80	16.70	N.D.	N.D.	N.D.	5.02	12.50	6.68	15.84 ± 28.93
	24	99.90	N.D.	6.50	12.20	24.40	5.03	39.70	1.84	23.70 ± 33.55
	36	37.90	N.D.	N.D.	N.D.	N.D.	N.D.	9.08	N.D.	5.87 ± 13.33
	48	61.40	N.D.	N.D.	N.D.	4.53	12.20	31.90	N.D.	13.75 ± 22.18
	60	27.80	4.19	N.D.	15.10	N.D.	N.D.	31.00	N.D.	9.76 ± 13.19
	72	N.D.	N.D.	N.D.	N.D.	N.D.	N.D.	19.60	N.D.	2.45 ± 6.93

Footnote: N.D. = not detected; SD = standard deviation.
